# Cellular Mechanisms Underlying Endothelial and Histopathological Alterations Induced by Cerebral Angiography

**DOI:** 10.3390/jcm15030974

**Published:** 2026-01-25

**Authors:** Zülfikar Özgür Ertuğrul, Mehmet Cudi Tuncer, Mehmet Uğur Karabat

**Affiliations:** 1 Department of Radiology, Gazi Yaşargil Training and Research Hospital, University of Health Sciences, Diyarbakır 21090, Turkey; stenoz@hotmail.com; 2 Department of Anatomy, Faculty of Medicine, Dicle University, Diyarbakir 21090, Turkey; 3 Department of Histology and Embryology, Medical Faculty, Dicle University, Diyarbakır 21090, Turkey; ugurkrbt@hotmail.com

**Keywords:** cerebral angiography, endothelial dysfunction, histopathological changes, blood–brain barrier, neurovascular injury

## Abstract

**Background/Objectives**: Cerebral angiography is a cornerstone diagnostic and therapeutic procedure for cerebrovascular diseases; however, its potential effects on vascular integrity and cellular homeostasis remain incompletely elucidated. This systematic review aims to comprehensively evaluate endothelial and histopathological alterations induced by cerebral angiographic procedures, with particular emphasis on oxidative stress, inflammation, endothelial dysfunction, and blood–brain barrier disruption. **Methods**: This systematic review was conducted in accordance with the PRISMA 2020 guidelines. PubMed, Scopus, and Web of Science databases were systematically searched for studies published between 1981 and 2025 using predefined keywords related to cerebral angiography, endothelial injury, oxidative stress, inflammation, and histopathological changes. A total of 1142 records were identified, and 216 duplicates were removed. Following title and abstract screening, 312 full-text articles were assessed for eligibility, of which 112 were excluded due to irrelevance or insufficient endothelial or histopathological data. Ultimately, 200 studies were included in the qualitative synthesis. The literature identification, screening, and selection process are summarized in the manuscript. The review protocol was not prospectively registered. **Results**: The included studies demonstrated that cerebral angiographic procedures induce endothelial and microvascular alterations through both mechanical and contrast-mediated mechanisms. Iodinated contrast agents were consistently associated with increased reactive oxygen species production, reduced endothelial nitric oxide bioavailability, mitochondrial dysfunction, and activation of pro-inflammatory signaling pathways, including nuclear factor kappa B (NF-κB). Histopathological findings revealed endothelial swelling, vacuolization, apoptosis, microthrombus formation, inflammatory cell infiltration, and disruption of endothelial junctions, leading to increased vascular permeability and blood–brain barrier impairment. Mechanical factors related to catheter manipulation and high-pressure contrast injection further exacerbated endothelial injury by altering shear stress and promoting leukocyte adhesion. The severity of endothelial damage and inflammatory responses was consistently greater in patients with comorbid conditions such as diabetes mellitus, hypertension, and atherosclerotic disease. **Conclusions**: Cerebral angiography may induce endothelial dysfunction and histopathological vascular injury predominantly through oxidative and inflammatory mechanisms. Optimization of contrast agent selection, refinement of procedural techniques, and implementation of endothelial-protective strategies may mitigate vascular injury and improve procedural safety. Further translational and clinical studies are warranted to identify biomarkers and protective interventions targeting angiography-induced endothelial damage.

## 1. Introduction

Cerebral angiography remains a cornerstone in the diagnosis and management of cerebrovascular diseases, including aneurysms, arteriovenous malformations (AVMs), and ischemic stroke. As an essential imaging modality, it provides detailed visualization of cerebral vasculature through the administration of contrast agents and fluoroscopic guidance. Despite its diagnostic value, cerebral angiography is associated with various biological and histopathological effects on endothelial cells and the vascular microenvironment. Understanding these changes is critical for mitigating potential risks and optimizing patient outcomes [1,2]. The inception of cerebral angiography dates back to the early 20th century, with the pioneering work of Egas Moniz in 1927, marking a revolutionary advancement in neurovascular imaging [3]. Over the decades, technological innovations, including digital subtraction angiography (DSA), have significantly improved image resolution and reduced procedural risks. Nonetheless, despite these advancements, the technique is not devoid of complications, particularly at the cellular and histopathological levels [4].

Endothelium, a monolayer of cells lining the interior of blood vessels, plays a fundamental role in maintaining vascular homeostasis, regulating blood flow, preventing thrombosis, and modulating inflammatory responses. The integrity of the endothelial barrier is crucial for preserving cerebral hemodynamics and preventing pathological states such as blood–brain barrier (BBB) disruption and vascular inflammation [5,6]. Any external insult, including contrast-induced endothelial dysfunction, can lead to transient or permanent histopathological alterations, affecting cerebrovascular health [7,8].

Cerebral angiography involves high-pressure contrast injections, inducing abrupt hemodynamic shear stress on endothelial cells. Such mechanical forces can lead to endothelial activation, increasing the expression of adhesion molecules and cytokines that promote vascular inflammation and dysfunction [9,10,11]. Iodinated contrast agents are essential for cerebral angiography but can induce oxidative stress, apoptosis, and transient permeability changes in the endothelium [12,13]. Contrast media have been implicated in endothelial nitric oxide synthase (eNOS) dysfunction, leading to vasoconstriction and compromised cerebrovascular regulation [14,15,16].

The exposure of endothelial cells to contrast agents can activate nuclear factor kappa B (NF-κB) and other pro-inflammatory pathways, resulting in cytokine release and leukocyte adhesion. Chronic or excessive inflammation may contribute to endothelial injury, subendothelial matrix remodeling, and possible long-term vascular damage. Histological studies have demonstrated endothelial swelling, microthrombosis, and focal disruptions in the vascular architecture following cerebral angiography. These changes can exacerbate existing cerebrovascular pathologies and potentially contribute to delayed neurological deficits [17,18,19,20].

The transient or persistent endothelial dysfunction associated with cerebral angiography has significant clinical implications. Contrast-induced neurotoxicity, increased risk of thrombosis, and BBB disruption may elevate the likelihood of post-procedural complications such as stroke, transient ischemic attack, and cognitive impairment. Understanding these risks underscores the importance of optimizing procedural techniques, contrast media selection, and potential protective strategies [21,22,23]. Recent advancements in molecular imaging and in vitro endothelial models offer promising avenues for exploring the cellular impact of cerebral angiography [24,25]. Future studies should focus on identifying biomarkers for contrast-induced endothelial dysfunction and evaluating protective pharmacological agents to mitigate vascular injury. The integration of nanotechnology and alternative contrast agents may also present novel strategies to enhance safety and efficacy in neuroangiographic procedures.

Cerebral angiography, while indispensable for diagnosing cerebrovascular diseases, induces a spectrum of endothelial and histopathological changes that warrant careful consideration. A deeper understanding of the cellular mechanisms underlying these alterations will facilitate the development of targeted interventions to minimize vascular injury and improve patient outcomes. This review aims to provide a comprehensive overview of the endothelial and histopathological changes induced by cerebral angiography, highlighting the need for further research into protective strategies and improved imaging techniques.

## 2. Materials and Methods

This systematic review was conducted following the PRISMA 2020 guidelines. Literature searches were performed using the PubMed, Scopus, and Web of Science databases, encompassing studies published between 1981 and 2025. Keywords included cerebral angiography, endothelial injury, and histopathology.

Studies were included if they evaluated endothelial dysfunction, oxidative stress, inflammatory markers, vascular permeability, or histopathological changes following angiographic procedures. Duplicates were removed, and titles and abstracts were screened for relevance. Full texts were assessed based on inclusion criteria, and non-cerebrovascular or non-histopathological studies were excluded.

The literature review and article selection process was conducted by three authors with complementary expertise. Prof. Dr. Mehmet Cudi Tuncer (anatomist), Dr. Mehmet Uğur Karabat (histologist), and Dr. Zülfikar Özgür Ertuğrul (radiologist) independently evaluated studies relevant to their respective fields. Each reference was primarily assessed by the author whose expertise best matched the study content (e.g., cellular and histopathological studies by anatomist and histologist; imaging- and angiography-related studies by the radiologist). Subsequently, all included studies, thematic categorizations, and interpretations were discussed collectively among the authors, and final decisions were reached through consensus and multidisciplinary scientific discussion.

The final synthesis comprised 200 eligible studies. The literature identification, screening, and inclusion process is summarized in Table 1 (PRISMA 2020 framework). This systematic review was conducted and reported in accordance with the Preferred Reporting Items for Systematic Reviews and Meta-Analyses (PRISMA) 2020 statement. A completed PRISMA 2020 flow diagram is provided in the Supplementary Materials (Figure S1). The review protocol was not prospectively registered.

## 3. Fundamentals of Cerebral Angiography and Endovascular Approaches

### 3.1. Digital Subtraction Angiography (DSA)

DSA provides detailed visualization of the vascular system by digitally subtracting a pre-contrast mask image from post-contrast images, thereby eliminating background structures and enabling selective depiction of blood vessels [26,27]. This technique requires arterial catheterization and intra-arterial contrast injection, which distinguishes DSA from non-invasive vascular imaging modalities and underlies its unique impact on the vascular endothelium.

The DSA procedure involves catheter-based arterial access and contrast injection, which may be associated with procedure-related complications such as bleeding, contrast-induced nephrotoxicity, or allergic reactions [28,29]. DSA plays a critical role in the diagnosis and management of neurovascular, cerebrovascular, and cardiovascular diseases, including the evaluation of cerebral aneurysms, arteriovenous malformations, ischemic stroke, arterial stenosis, and various peripheral vascular pathologies [30,31,32,33,34,35]. Its high spatial and temporal resolution allows detailed visualization of vascular structures and facilitates interventional procedures with lower contrast volumes compared to conventional angiography [36,37]. However, the invasive nature of DSA increases the risk of complications such as vascular injury, embolic events, radiation exposure, and contrast-related adverse effects, particularly in patients with pre-existing comorbidities [38,39]. Recent technological advancements, including flat-panel detector systems and three-dimensional rotational angiography, have improved image quality and procedural precision while reducing radiation exposure [40,41,42]. Despite these developments, DSA remains the gold standard among vascular imaging modalities owing to its superior spatial and temporal resolution and real-time dynamic imaging capabilities [43,44]. 

In conclusion, DSA remains the gold standard for diagnosing vascular diseases and guiding interventional procedures. With continuous advancements in imaging technology and low-dose protocols, its safety and effectiveness have improved. Future developments, such as AI-assisted analysis and hybrid imaging techniques, are expected to further expand its clinical applications, making it an even more valuable tool in minimally invasive treatment approaches (Table 2).

### 3.2. Magnetic Resonance Angiography (MRA)

Magnetic resonance angiography (MRA) is a non-invasive vascular imaging modality that enables visualization of cerebral vessels without arterial catheterization or exposure to ionizing radiation [45,46,47]. The absence of intravascular instrumentation eliminates direct mechanical endothelial injury, distinguishing MRA from catheter-based angiographic techniques. Accordingly, MRA is generally associated with a lower risk of procedure-related endothelial disruption and histopathological vascular damage compared with digital subtraction angiography [48,49,50].

Although many MRA techniques can be performed without contrast agents, contrast-enhanced MRA involves the intravenous administration of gadolinium-based agents, which have been associated with endothelial alterations under specific conditions [51,52,53,54]. Experimental and clinical data suggest that gadolinium exposure may contribute to endothelial dysfunction through oxidative stress pathways and impaired vascular integrity, particularly in patients with reduced renal clearance [55,56,57,58,59,60]. In addition, MRA-derived hemodynamic parameters enable indirect assessment of endothelial shear stress, a critical determinant of vascular remodeling and atherosclerotic progression. Altered shear stress patterns identified by MRA have been linked to endothelial dysfunction and dysregulated nitric oxide and vascular endothelial growth factor signaling, highlighting the role of MRA as a non-invasive tool for studying cerebrovascular endothelial biology rather than a direct cause of endothelial injury [61,62,63,64,65,66].

MRA has revolutionized cerebrovascular imaging by offering a non-invasive, radiation-free alternative to conventional angiography. While MRA minimizes direct endothelial damage, its ability to assess hemodynamic alterations and endothelial function plays a critical role in understanding cerebrovascular pathophysiology. Ongoing technological advancements in MRA will continue to refine its diagnostic accuracy, making it an invaluable tool for studying vascular diseases and their cellular implications (Table 3).

**Figure 1:**
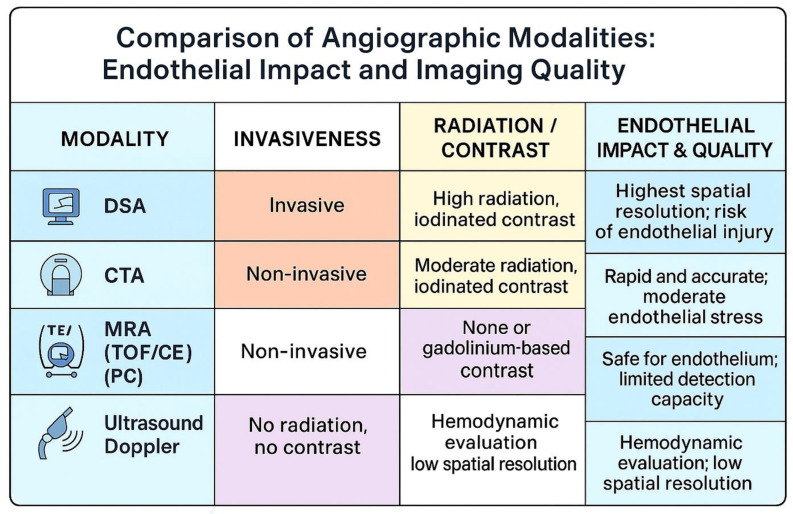
Comparison of angiographic modalities based on invasiveness, radiation exposure, contrast requirements, and endothelial impact. This infographic compares the primary angiographic techniques—DSA, CTA, MRA (MRA; TOF, CE, PC), and ultrasound Doppler—in terms of invasiveness, imaging quality, and endothelial safety. DSA provides the highest spatial resolution but carries the greatest risk of endothelial injury due to high radiation and iodinated contrast exposure. CTA offers rapid image acquisition with moderate radiation and contrast use, posing moderate endothelial stress. MRA, employing TOF, CE, or PC sequences, is non-invasive and safe for the endothelium, using gadolinium-based contrast or none at all, though it has limited calcification detection. Ultrasound Doppler is completely radiation- and contrast-free, suitable for hemodynamic evaluation but with lower spatial resolution compared to the other modalities.

The comparative visualization of angiographic techniques is illustrated in Figure 1.

### 3.3. Computed Tomography Angiography (CTA)

CTA utilizes multidetector computed tomography with intravenous iodinated contrast administration to visualize the cerebrovascular system with high spatial resolution. As a non-invasive technique that does not require arterial catheterization, CTA avoids direct mechanical endothelial injury associated with conventional angiography. However, the use of iodinated contrast agents and exposure to ionizing radiation introduce potential risks that are relevant in the context of angiography-related endothelial and histopathological injury [67,68,69,70,71].

From a cellular perspective, iodinated contrast agents have been shown to induce endothelial dysfunction through multiple mechanisms, including increased oxidative stress, inflammatory activation, and disruption of endothelial barrier integrity. Experimental and clinical studies suggest that contrast exposure may alter endothelial permeability, impair nitric oxide bioavailability, and promote the expression of pro-inflammatory cytokines such as tumor necrosis factor-α and interleukin-6, contributing to endothelial activation and microvascular injury. These effects may be more pronounced in patients with pre-existing vascular risk factors, including diabetes mellitus and hypertension [72,73,74,75,76,77,78].

Radiation exposure represents an additional consideration associated with CTA. Although contemporary low-dose protocols and reconstruction techniques have substantially reduced radiation burden, cumulative exposure may still contribute to long-term vascular and tissue effects, particularly in patients requiring repeated imaging [74,75,79,80]. Compared with other non-invasive modalities, CTA provides rapid acquisition and high spatial resolution but lacks the advantage of radiation-free imaging [81,82,83].

Overall, CTA offers rapid and accurate cerebrovascular assessment while minimizing the mechanical endothelial injury inherent to catheter-based angiography. Nevertheless, contrast-induced endothelial dysfunction and radiation exposure remain relevant concerns, underscoring the importance of protocol optimization and careful patient selection. Understanding the endothelial and histopathological consequences of contrast exposure in CTA remains essential for minimizing vascular injury in clinical practice [84,85].

### 3.4. Intraoperative Angiographic Techniques

Intraoperative angiographic techniques play a critical role in neurosurgery, vascular surgery, and interventional radiology by providing real-time visualization of cerebral vasculature during surgical procedures. These techniques allow for immediate assessment of vascular anatomy, identification of intraoperative complications, and confirmation of successful surgical interventions. Intraoperative angiography is most commonly utilized in procedures such as aneurysm clipping, AVMs resection, extracranial-to-intracranial (EC-IC) bypass surgery, and endovascular interventions, where precise vascular assessment is essential for optimizing patient outcomes. Unlike preoperative angiographic imaging modalities such as CTA or MRA, intraoperative techniques provide direct feedback during surgery, reducing the need for revision procedures and improving surgical accuracy [86,87].

The primary intraoperative angiographic modalities include intraoperative digital subtraction angiography (iDSA), indocyanine green (ICG) angiography, intraoperative Doppler ultrasonography, and intraoperative fluorescence imaging. Each of these techniques offers distinct advantages and limitations, depending on the surgical setting and the vascular pathology being treated [88,89].

iDSA is considered the gold standard for intraoperative vascular imaging. This technique involves the use of an intraoperative C-arm fluoroscopic system or a dedicated biplane angiography suite to acquire high-resolution images of the cerebral vasculature. A catheter is introduced into the arterial system, typically via the femoral artery, and contrast media is injected to enhance vascular structures. Subtraction algorithms are then applied to remove background noise, allowing for clear visualization of the blood vessels. iDSA is particularly useful in complex neurosurgical procedures, such as aneurysm clipping, where precise assessment of vessel patency, residual aneurysm filling, or unintended vessel occlusion is critical. Studies have demonstrated that iDSA can detect remnant aneurysmal necks that are not visible under the operating microscope, leading to immediate surgical correction and improved patient outcomes. However, the use of iDSA is associated with certain drawbacks, including the need for an angiography-capable operating room, increased procedural time, exposure to ionizing radiation, and potential complications related to arterial catheterization [90,91,92].

ICG angiography has gained widespread use as a non-invasive intraoperative imaging modality for assessing cerebral blood flow. ICG is a fluorescent dye that binds to plasma proteins and remains confined to the vascular compartment. When illuminated with near-infrared (NIR) light, the dye fluoresces, allowing for real-time visualization of blood flow under the surgical microscope. ICG angiography is particularly advantageous in aneurysm surgery, as it provides immediate confirmation of aneurysm exclusion and adjacent vessel patency without the need for additional contrast injections or radiation exposure. Additionally, ICG angiography is widely used in AVM and dAVF surgery, where it helps delineate feeding arteries and draining veins. Compared to iDSA, ICG angiography is faster, safer, and easier to perform. However, its primary limitation is its inability to visualize deep-seated or overlapping vessels, as the fluorescence signal is limited to the superficial cortical structures [93,94].

Intraoperative Doppler ultrasonography serves as an adjunct to other angiographic techniques for assessing blood flow velocity and vessel patency during cerebrovascular surgery. This modality utilizes pulsed-wave and color Doppler imaging to detect flow disturbances, stenosis, or occlusions in real-time. Doppler ultrasonography is particularly useful in EC-IC bypass surgery, where it allows for the immediate evaluation of bypass graft function and anastomotic integrity. The technique is non-invasive, does not require contrast administration, and is free of radiation exposure. However, its main limitations include operator dependency, limited spatial resolution, and difficulty in assessing deep vascular structures [95,96].

Intraoperative fluorescence imaging techniques, such as fluorescein angiography and sodium fluorescein-enhanced video angiography, provide an alternative method for visualizing cerebral vasculature. Fluorescein angiography utilizes sodium fluorescein dye, which emits a yellow-green fluorescence under blue light excitation. This technique has been used to assess BBB integrity and cerebral perfusion during tumor resection and vascular procedures. However, fluorescein angiography has largely been replaced by ICG angiography due to its superior image quality and higher vascular specificity [97,98].

The impact of intraoperative angiographic techniques on endothelial integrity and histopathological changes in the cerebral vasculature remains an area of ongoing research. While these techniques offer critical advantages in surgical precision and vascular assessment, they may also induce cellular alterations due to contrast exposure, mechanical manipulation, and radiation effects. iDSA, for instance, involves the use of iodinated contrast agents, which have been shown to cause endothelial dysfunction through oxidative stress, inflammation, and alterations in NO signaling. The hyperosmolar nature of contrast media can lead to endothelial cell shrinkage, increased permeability, and activation of pro-inflammatory pathways involving TNF-α and IL-6. Additionally, prolonged exposure to radiation during iDSA may contribute to endothelial DNA damage and cellular apoptosis, particularly in prolonged or repeated procedures [99,100,101].

ICG angiography, while generally considered safe, has been associated with transient hemodynamic alterations due to its interaction with plasma proteins. Some studies suggest that ICG may induce mild endothelial stress, particularly in patients with pre-existing vascular pathology. However, these effects are typically transient and do not result in long-term endothelial injury. In contrast, Doppler ultrasonography and fluorescence imaging techniques do not involve contrast agents or ionizing radiation, making them less likely to induce histopathological changes. Nevertheless, mechanical manipulation of vessels during ultrasound probe application may exert localized pressure on the endothelium, potentially leading to endothelial shear stress and minor disruptions in vascular integrity [102,103]. Although ICG angiography is considered highly safe, theoretical concerns exist that, in patients with compromised vascular integrity, transient endothelial stress may occur due to its plasma protein interactions; however, no direct evidence of long-term endothelial injury has been demonstrated in the literature.

The selection of an appropriate intraoperative angiographic technique depends on multiple factors, including the type of cerebrovascular pathology, the surgical approach, and the need for real-time feedback. While iDSA remains the most comprehensive method for intraoperative vascular imaging, its invasiveness, radiation exposure, and procedural complexity limit its routine use. ICG angiography has emerged as a preferred alternative due to its simplicity, real-time imaging capability, and safety profile. Doppler ultrasonography and fluorescence imaging serve as valuable adjuncts, particularly in procedures requiring hemodynamic assessment [104,105].

Future advancements in intraoperative angiography aim to enhance image resolution, reduce invasiveness, and minimize endothelial and histopathological alterations. Emerging technologies such as intraoperative optical coherence tomography offer the potential for high-resolution, label-free imaging of cerebral vessels at the microscopic level. AI-assisted image analysis is also being integrated into intraoperative workflows, enabling automated vessel segmentation, flow quantification, and real-time surgical guidance. Additionally, novel contrast agents with improved biocompatibility and reduced endothelial toxicity are under investigation.

Intraoperative angiographic techniques have significantly improved the safety and efficacy of cerebrovascular surgery by enabling real-time vascular assessment and immediate correction of intraoperative complications. While these techniques offer substantial benefits, understanding their potential impact on endothelial function and histopathology is essential for optimizing their application. Continued research into the cellular effects of intraoperative imaging modalities will contribute to the development of safer and more effective techniques for vascular visualization in neurosurgical practice.

## 4. Effects of Angiographic Procedures on Cerebral Arteries

### 4.1. Endothelial Cell Damage

Endothelial cells form a critical monolayer lining the inner surface of blood vessels, maintaining vascular homeostasis, regulating permeability, and modulating hemodynamic forces. During cerebral angiography, endothelial integrity may be compromised by mechanical, chemical, and radiation-related insults, resulting in functional and structural alterations. Histological evaluation of endothelial cell damage provides essential insight into angiography-associated pathophysiological processes, including inflammation, oxidative stress, and vascular remodeling [106,107,108].

**Figure 2:**
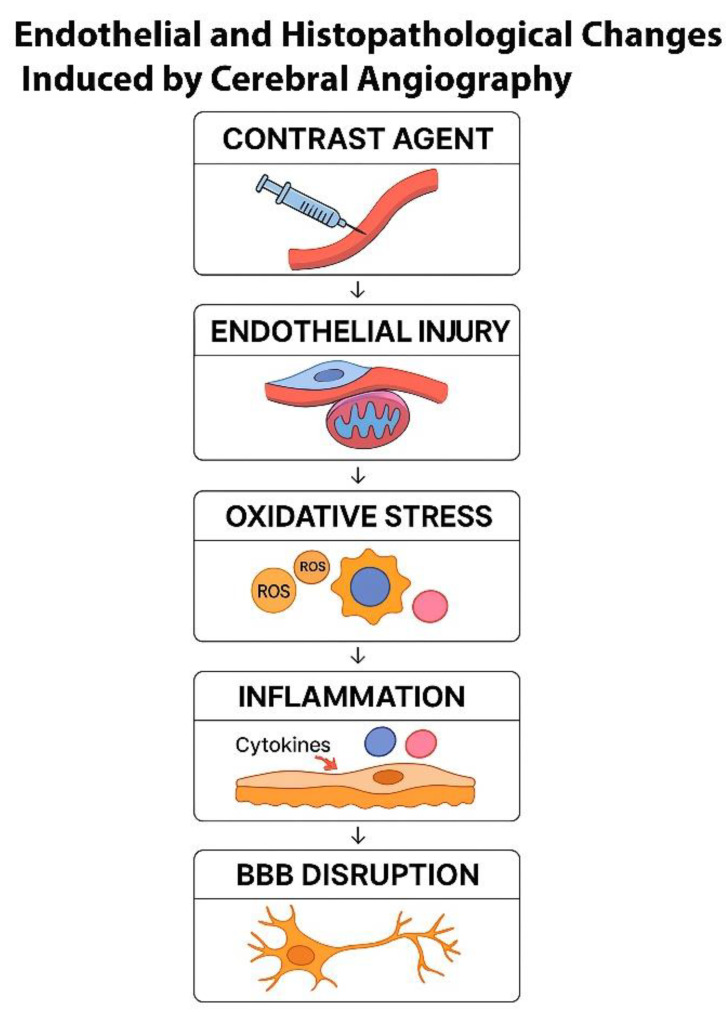
Schematic representation of the sequential pathophysiological mechanisms triggered by cerebral angiography. The process begins with the intravascular administration of a contrast agent, which interacts with the vascular endothelium and induces cellular stress. Subsequent endothelial injury compromises membrane integrity and mitochondrial function, promoting oxidative stress through excessive generation of ROS such as superoxide anion (O_2_^−^), hydroxyl radical (•OH), and hydrogen peroxide (H_2_O_2_). Accumulated ROS activate inflammatory signaling, characterized by the release of pro-inflammatory cytokines including TNF-α, IL-6, and interleukin-1 beta (IL-1β). These cytokines disrupt endothelial junctions and increase vascular permeability, resulting in BBB breakdown and subsequent neuronal dysfunction. The diagram illustrates this cascade as: Contrast Agent → Endothelial Injury → Oxidative Stress → Inflammation → BBB Disruption.

The interplay between oxidative stress and inflammation is a major contributor to endothelial dysfunction and subsequent BBB breakdown (Figure 2).

**Figure 3:**
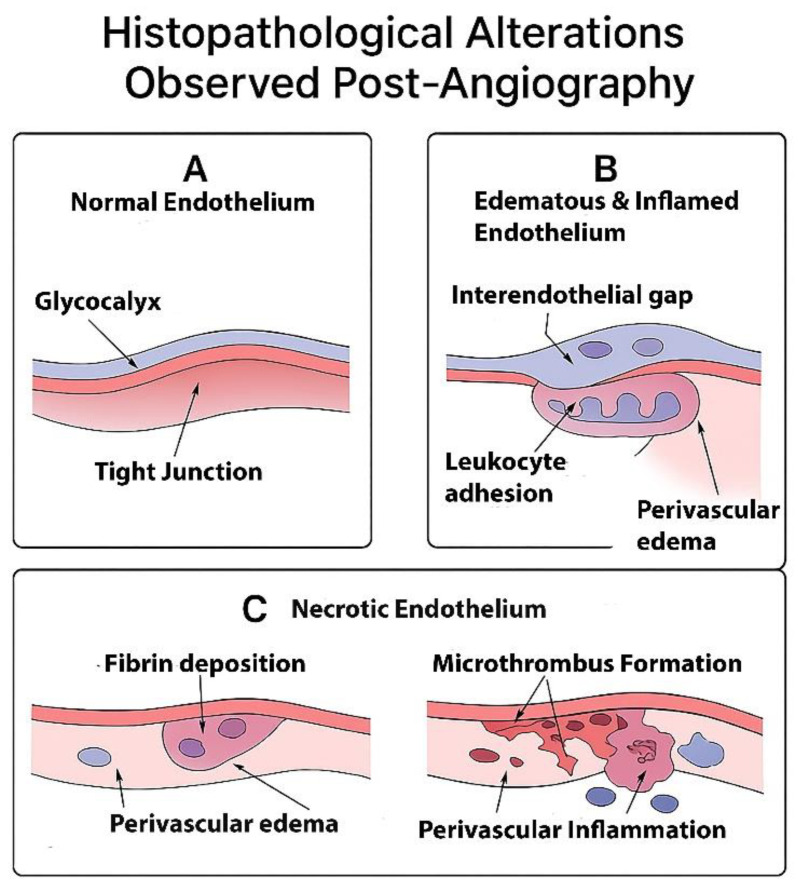
Schematic illustration depicting sequential endothelial and perivascular changes following cerebral angiography. Panel (**A**) shows normal endothelium with intact glycocalyx and tight junctions. Panel (**B**) illustrates edematous and inflamed endothelium with endothelial swelling, interendothelial gap formation, leukocyte adhesion, and perivascular edema. Panel (**C**) presents necrotic endothelium characterized by fibrin deposition, microthrombus formation, perivascular inflammation, and perivascular edema, representing advanced vascular injury induced by contrast exposure.

Histological examination of endothelial damage typically involves hematoxylin and eosin staining, immunohistochemical markers, and ultrastructural analysis using electron microscopy. Light microscopy of affected cerebral vessels reveals endothelial cell swelling, vacuolation, disruption of normal endothelial architecture, and vascular congestion. These alterations are frequently associated with increased vascular permeability, as evidenced by extravasation of plasma proteins such as fibrinogen and albumin into the perivascular space. In more severe cases, endothelial denudation with exposure of the underlying basement membrane and intimal layer may be observed, predisposing the vessel wall to thrombogenic activity and microthrombus formation [109,110]. Representative histopathological features observed following cerebral angiography are illustrated in Figure 3.

Immunohistochemical markers are essential for evaluating endothelial injury at the molecular level. Vascular endothelial cadherin and platelet endothelial cell adhesion molecule-1 are commonly used to assess intercellular junction integrity, with loss of VE-cadherin staining indicating barrier disruption and upregulation of PECAM-1 reflecting endothelial activation and leukocyte transmigration. Endothelial nitric oxide synthase expression is frequently reduced in injured endothelium, consistent with impaired nitric oxide–mediated vasoregulation. In parallel, markers of oxidative stress, including 3-nitrotyrosine and malondialdehyde, are often elevated, reflecting peroxynitrite-mediated oxidative injury [111,112,113].

Transmission electron microscopy provides detailed insight into endothelial ultrastructural alterations associated with angiography-related injury. Common findings include endothelial cell detachment, mitochondrial swelling, chromatin condensation, and intracellular vacuolization, consistent with apoptotic and metabolic stress responses. Endothelial microparticles shed from activated or apoptotic endothelial cells are frequently observed within the vascular lumen and serve as indicators of endothelial injury and systemic vascular stress [114,115,116].

Histopathological endothelial damage may be further exacerbated by contrast-induced toxicity. Iodinated contrast agents used in angiographic procedures have been shown to induce endothelial apoptosis through reactive oxygen species generation and mitochondrial dysfunction. In addition, contrast media can disrupt the endothelial glycocalyx, a critical regulator of vascular permeability, which can be demonstrated by lectin-based staining and ultrastructural imaging techniques [16,100,117].

Radiation exposure during cerebral angiography represents an additional injurious stimulus to the vascular endothelium. Ionizing radiation induces DNA strand breaks and activates pro-apoptotic signaling pathways, as evidenced by increased expression of γ-H2AX, p53, and caspase-3. Chronic or repeated exposure may promote endothelial senescence, characterized by increased β-galactosidase activity and upregulation of p16INK4a and p21, thereby impairing vascular repair capacity [118,119,120].

Beyond direct cellular injury, endothelial damage initiates secondary inflammatory and thrombotic responses. Increased expression of endothelial adhesion molecules such as intercellular adhesion molecule-1 and vascular cell adhesion molecule-1 facilitates leukocyte adhesion and transmigration, accompanied by infiltration of monocytes and neutrophils and elevated levels of pro-inflammatory cytokines including TNF-α and IL-6. These processes contribute to sustained endothelial dysfunction, vascular remodeling, and an increased risk of post-procedural thrombosis [121,122,123].

Histological assessment of endothelial repair mechanisms following angiographic injury demonstrates activation of endothelial progenitor cells, identified by CD34 and vascular endothelial growth factor receptor-2 expression. However, in the setting of severe or repetitive injury, reparative responses may be insufficient, resulting in persistent endothelial dysfunction and increased susceptibility to atherosclerotic plaque development [124,125].

### 4.2. Vascular Wall Inflammation and Immunological Response

Cerebral angiographic procedures can induce vascular wall inflammation and immunological responses that contribute to endothelial dysfunction, thrombosis, and restenosis. These processes are initiated by endothelial injury resulting from mechanical stress during catheter manipulation, alterations in shear stress, and the cytotoxic effects of iodinated contrast agents. Endothelial activation is characterized by increased vascular permeability, oxidative stress, and upregulation of adhesion molecules that facilitate leukocyte recruitment [126,127].

Following endothelial injury, an inflammatory cascade is triggered, involving the release of pro-inflammatory cytokines such as TNF-α, IL-6, and IL-1β. Activation of the innate immune system leads to neutrophil infiltration and the release of reactive oxygen species and proteolytic enzymes, further exacerbating vascular injury. Monocytes and macrophages contribute to sustained inflammation through mediator release and clearance of apoptotic endothelial cells. In addition, complement activation amplifies immune responses and vascular permeability, while adaptive immune mechanisms, including CD4+ T-cell activation, may perpetuate chronic inflammation following exposure of endothelial antigens [128,129].

The clinical consequences of angiography-associated vascular inflammation include an increased risk of thrombosis, restenosis, and disruption of the blood–brain barrier, potentially resulting in neurological complications. Systemic inflammatory responses may also contribute to contrast-induced nephropathy in susceptible patients. Strategies aimed at mitigating these effects include minimizing endothelial trauma, optimizing contrast dose, and improving procedural biocompatibility, as well as appropriate pre-procedural patient optimization in high-risk populations.

### 4.3. Effects on the Blood–Brain Barrier

Cerebral angiographic procedures can affect blood–brain barrier integrity through mechanical, chemical, and inflammatory mechanisms. Endothelial injury induced by catheter manipulation, fluctuations in shear stress during contrast injection, and procedure-related inflammatory responses may collectively compromise barrier function and contribute to angiography-associated vascular injury [110,130,131,132,133].

Endothelial injury represents a primary mechanism underlying BBB disruption during angiographic procedures. Mechanical stress associated with catheter insertion and navigation, together with shear stress alterations, can impair endothelial integrity, increase paracellular permeability, and expose the underlying basement membrane, thereby promoting inflammatory activation and vascular injury [134,135,136].

Contrast agents used during angiography further contribute to BBB alterations. Iodinated contrast media commonly employed in DSA can induce oxidative stress and cytotoxic effects on endothelial cells, leading to disruption of tight junction proteins, increased permeability, and endothelial apoptosis. Hyperosmolar contrast agents may exacerbate these effects by directly impairing endothelial junctional integrity and facilitating extravasation of inflammatory mediators and immune cells into the brain parenchyma [133,137,138].

Procedure-related inflammatory responses can further amplify BBB dysfunction. Endothelial activation promotes the release of pro-inflammatory cytokines, including TNF-α, IL-6, and IL-1β, which enhance leukocyte adhesion and transmigration within the cerebral microvasculature. This inflammatory cascade weakens barrier integrity and increases the risk of neuroinflammation, cerebral edema, and microvascular thrombosis [139,140,141].

Clinically, BBB disruption following cerebral angiography has been associated with neurological complications such as contrast-induced neurotoxicity, transient ischemic events, and increased susceptibility to hemorrhagic transformation in vulnerable patients. Strategies aimed at mitigating BBB injury include optimization of procedural techniques, minimization of catheter manipulation and contrast volume, and appropriate patient optimization in high-risk populations [130,137].

## 5. Histopathological Effects of Angiographic Contrast Agents

### 5.1. Endothelial Dysfunction and Oxidative Stress

Histopathological and experimental studies have demonstrated that endothelial dysfunction and oxidative stress represent key cellular consequences of cerebral angiographic procedures. Rather than redefining mechanistic pathways, this section focuses on the histological and molecular manifestations of angiography-associated endothelial injury observed at the tissue and cellular levels.

Oxidative stress–related endothelial damage is characterized by increased production of reactive oxygen species, leading to lipid peroxidation, protein oxidation, and mitochondrial dysfunction within endothelial cells. Histological and molecular analyses have shown increased endothelial apoptosis, impaired nitric oxide bioavailability, and disruption of endothelial barrier integrity following exposure to contrast media and procedural stress. These alterations are further exacerbated in settings of ischemia–reperfusion, where endothelial cells exhibit heightened susceptibility to oxidative injury due to mitochondrial dysfunction and reduced antioxidant capacity [5,16,78,99].

At the vascular level, endothelial dysfunction manifests as impaired vasoregulatory responses, increased platelet adhesion, and a prothrombotic endothelial phenotype. Experimental models and clinical observations have reported upregulation of inflammatory mediators, adhesion molecules, and oxidative stress markers within cerebral vessels following angiographic procedures. These changes correlate with histopathological findings such as endothelial swelling, microvascular congestion, and focal ischemic injury, providing structural evidence of functional endothelial impairment [131,137,142].

Collectively, the histopathological evidence underscores the close association between endothelial dysfunction, oxidative stress, and angiography-related vascular injury. These cellular alterations contribute to post-procedural complications including cerebral ischemia, vasospasm, and contrast-induced neurotoxicity, highlighting the importance of preventive strategies aimed at minimizing endothelial stress during cerebral angiographic procedures.

### 5.2. Contrast-Induced Nephropathy and Neurovascular Toxicity

Iodinated contrast media used during cerebral angiographic procedures may induce systemic toxic effects, most notably contrast-induced nephropathy and neurovascular toxicity. From a cellular and histopathological perspective, contrast exposure is associated with endothelial dysfunction, oxidative stress, and microvascular injury in both renal and cerebral tissues.

In the kidney, contrast-induced nephropathy is characterized by renal microvascular vasoconstriction, medullary hypoxia, and direct tubular epithelial toxicity. Experimental and histopathological studies have demonstrated increased oxidative stress, mitochondrial dysfunction, and tubular cell apoptosis following contrast exposure. These effects are mediated by an imbalance between vasodilatory and vasoconstrictive signaling pathways, resulting in impaired renal perfusion and ischemic injury, particularly in susceptible individuals with pre-existing vascular or metabolic comorbidities [74,130,137,143].

Neurovascular toxicity represents a parallel manifestation of contrast-related injury within the central nervous system. Contrast agents may induce cerebral endothelial dysfunction, increased blood–brain barrier permeability, and vasogenic edema through hyperosmolar and cytotoxic effects on endothelial cells. Histopathological and imaging-based observations support the association between contrast exposure and transient neurovascular injury, including endothelial swelling, microvascular dysfunction, and reversible neuronal stress. These alterations are more frequently observed in patients with underlying cerebrovascular risk factors and are typically self-limited but clinically relevant [137,144].

Together, contrast-induced nephropathy and neurovascular toxicity underscore the systemic nature of contrast-mediated endothelial injury. These findings highlight the importance of minimizing contrast burden, optimizing procedural strategies, and identifying high-risk patients to reduce cellular and histopathological damage associated with cerebral angiographic procedures.

### 5.3. Cellular-Level Inflammation and Necrosis Mechanisms

At the cellular level, cerebral angiographic procedures are associated with inflammatory activation and cell death within the vascular and perivascular compartments. Histopathological and experimental studies demonstrate that endothelial injury during angiography is accompanied by increased production of pro-inflammatory cytokines, including TNF-α, IL-6, and IL-1β, which promote leukocyte recruitment and amplify local inflammatory responses. Infiltrating neutrophils and monocytes contribute to cellular injury through the generation of reactive oxygen and nitrogen species, thereby exacerbating endothelial dysfunction and tissue damage [14,17,108,112].

Contrast-related cytotoxicity represents a key contributor to cellular injury. Exposure to iodinated contrast media has been shown to induce oxidative stress, mitochondrial dysfunction, and disruption of cellular membrane integrity. These alterations promote lipid peroxidation, protein oxidation, and DNA damage, ultimately triggering apoptotic or necrotic cell death within endothelial and neural cells. Increased vascular permeability and blood–brain barrier disruption facilitate inflammatory cell infiltration into the brain parenchyma, further aggravating neuroinflammation [16,17,25,78,99].

Ischemia–reperfusion injury further intensifies cellular inflammation and necrosis during cerebral angiographic procedures. Transient reductions in cerebral blood flow associated with catheter manipulation or microembolic events induce hypoxic stress, leading to activation of hypoxia-responsive signaling pathways and inflammatory mediator release. Subsequent reperfusion results in excessive reactive oxygen species production, amplifying oxidative damage and promoting neuronal apoptosis or necrosis. Histopathological evidence supports the contribution of ischemia–reperfusion–related injury to angiography-associated tissue damage and neurological vulnerability [99,110].

In advanced stages of endothelial injury, activation of coagulation pathways and complement cascades may occur, resulting in microthrombus formation and direct cellular lysis. The interplay between endothelial cells, platelets, leukocytes, and complement components sustains a pro-inflammatory microenvironment that increases susceptibility to ischemic injury and tissue necrosis [16,17].

## 6. Secondary Vascular and Cellular Effects of Endovascular Therapeutic Procedures

### 6.1. Cellular-Level Effects of Endovascular Interventions

Endovascular interventions such as stenting, balloon angioplasty, and coil embolization are widely used for the treatment of cerebrovascular and peripheral vascular pathologies; however, these procedures are associated with distinct histopathological alterations of the vascular wall. Although they fall outside the primary scope of angiography-induced injury, their tissue-level effects merit brief consideration in the context of vascular endothelial damage.

Stent deployment induces acute endothelial denudation, exposing subendothelial structures and promoting platelet aggregation and thrombus formation. This is followed by inflammatory cell infiltration at the stent–vessel interface and progressive vascular remodeling. Smooth muscle cell proliferation and extracellular matrix deposition contribute to neointimal hyperplasia, which may result in in-stent restenosis. In later stages, chronic inflammation, neoatherosclerosis, and stent thrombosis have been reported, particularly in association with bare-metal stents [145,146,147,148].

Balloon angioplasty produces acute mechanical injury to the vessel wall, including endothelial disruption, intimal tearing, and variable medial layer damage. Histopathological findings commonly include elastic recoil, endothelial loss, and subsequent reparative responses characterized by endothelial regeneration, smooth muscle cell proliferation, and collagen deposition. These processes may culminate in neointimal thickening, with the severity of injury influenced by inflation pressure, balloon dimensions, and duration of dilation [149,150,151].

Coil embolization elicits a localized histopathological response dominated by thrombus formation within the aneurysmal sac, followed by inflammatory cell infiltration and progressive fibrosis. Platelet aggregation and fibrin deposition stabilize the thrombus in the early phase, while fibroblastic proliferation and collagen deposition contribute to aneurysm wall remodeling over time. Incomplete neointimal coverage of coils may permit recanalization, and persistent inflammatory reactions—particularly with non-bioresorbable coils—have been implicated in delayed aneurysm recurrence [106,152,153].

Collectively, these endovascular techniques induce procedure-specific histopathological changes that influence vascular healing, inflammatory responses, and long-term outcomes. A summary of the characteristic tissue-level effects associated with these interventions is provided in Table 4.

### 6.2. Cellular Changes Following Mechanical Thrombectomy

Mechanical thrombectomy is an established therapeutic intervention for acute ischemic stroke due to large vessel occlusion; however, the procedure is associated with distinct cellular and vascular alterations that may influence post-procedural recovery and complications. At the tissue level, mechanical retrieval of intravascular thrombi can result in direct endothelial injury, microvascular disruption, and activation of inflammatory and coagulation pathways [154,155,156].

Histopathological observations demonstrate endothelial denudation, subendothelial exposure, and focal vascular injury following thrombectomy. These changes promote platelet aggregation, thrombin activation, and local inflammatory responses, which may contribute to secondary thrombosis and vascular dysfunction. Reparative processes, including endothelial regeneration and vascular remodeling, are subsequently initiated but may be incomplete in the setting of extensive injury [157,158,159].

Mechanical thrombectomy is also associated with pronounced inflammatory and oxidative responses. Elevated levels of pro-inflammatory cytokines, leukocyte infiltration, and excessive reactive oxygen species production have been documented, particularly in the context of ischemia–reperfusion injury. Revascularization restores cerebral perfusion but simultaneously triggers oxidative stress, mitochondrial dysfunction, and neuronal apoptosis, thereby exacerbating endothelial and parenchymal injury [160,161,162,163].

In addition to vascular effects, cellular responses within the neurovascular unit are observed following thrombectomy. Activation of astrocytes and microglia contributes to neuroinflammatory signaling and may influence neuronal survival and synaptic remodeling. While glial activation can support tissue repair, excessive or sustained activation may amplify secondary injury and impair functional recovery [164,165,166,167].

Blood–brain barrier dysfunction represents a further consequence of thrombectomy-related cellular injury. Endothelial disruption, inflammatory mediator release, and oxidative stress increase barrier permeability, facilitating plasma protein extravasation and immune cell infiltration. These processes are associated with vasogenic edema and an increased risk of hemorrhagic transformation, which are critical determinants of post-thrombectomy outcomes. The principal cellular and histopathological changes observed following mechanical thrombectomy are summarized in Table 5 [168,169].

### 6.3. Restenosis and Tissue Regeneration Processes

Restenosis and tissue regeneration represent dynamic vascular responses following mechanical thrombectomy and contribute to long-term vascular and neurological outcomes. These processes are initiated by endothelial injury, inflammatory activation, and vascular remodeling occurring after successful recanalization [170].

Histopathological studies demonstrate that restenosis is primarily associated with endothelial dysfunction, smooth muscle cell proliferation, and extracellular matrix remodeling. Mechanical disruption of the endothelial layer promotes platelet activation and inflammatory cell recruitment, leading to neointimal hyperplasia and progressive luminal narrowing. Increased deposition of collagen and fibronectin, together with altered matrix metalloproteinase activity, contributes to vascular wall stiffening and pathological remodeling [171,172,173,174].

Concurrently, tissue regeneration mechanisms are activated to restore vascular integrity. Endothelial repair is supported by endothelial progenitor cell recruitment and pro-angiogenic signaling, while a shift toward anti-inflammatory and tissue-repair phenotypes within the inflammatory milieu facilitates vascular healing. These reparative processes aim to re-establish endothelial continuity and stabilize the vessel wall following injury [175,176,177].

At the neurovascular level, restoration of cerebral perfusion supports neuronal survival and promotes recovery within the neurovascular unit. Astrocytes, pericytes, and endothelial cells collectively contribute to barrier stabilization and vascular repair, while synaptic remodeling and axonal plasticity influence functional recovery. The principal histopathological features associated with restenosis and tissue regeneration following mechanical thrombectomy are summarized in Table 6.

## 7. Pathogenesis of Tissue Damage Following Cerebral Angiography

### 7.1. Ischemia–Reperfusion Injury and Cellular Response

Cerebral angiographic procedures may be associated with ischemia–reperfusion injury as a result of transient hypoperfusion, embolic phenomena, or prolonged catheter manipulation. Although often subclinical, these events can trigger cellular and vascular responses that contribute to angiography-related tissue injury. Experimental and clinical evidence implicates oxidative stress, inflammatory activation, excitotoxicity, blood–brain barrier dysfunction, and mitochondrial impairment as key contributors to ischemia–reperfusion–related damage in this setting [155,169].

Reperfusion is characterized by excessive generation of reactive oxygen species, leading to lipid peroxidation, protein oxidation, and DNA damage, which promote neuronal apoptosis and necrosis. Concurrent activation of microglia and astrocytes results in the release of pro-inflammatory cytokines, including TNF-α, IL-1β, and IL-6, further amplifying secondary injury through endothelial activation and leukocyte recruitment. Excitotoxic signaling, mediated by excessive glutamate release and calcium influx, exacerbates mitochondrial dysfunction and accelerates neuronal cell death. In parallel, endothelial injury and matrix metalloproteinase activation increase vascular permeability, contributing to vasogenic edema and microvascular instability [162,178,179].

**Figure 4:**
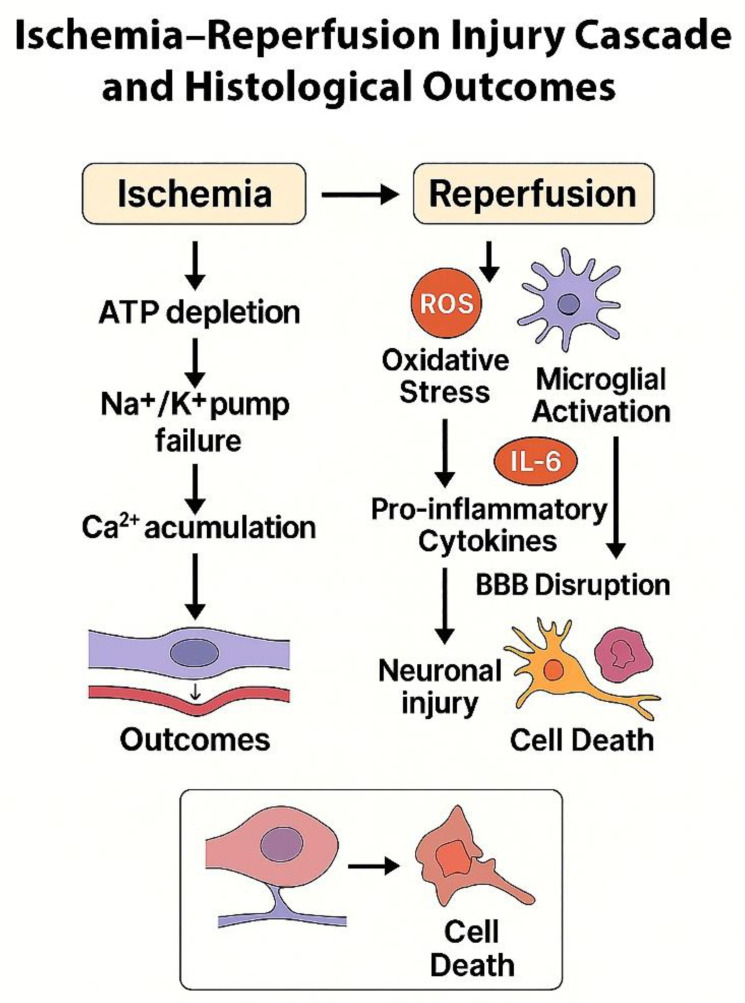
Schematic illustration depicting the sequential cellular and histopathological mechanisms underlying ischemia–reperfusion injury following cerebral angiography. The ischemic phase involves ATP depletion, Na^+^/K^+^ pump failure, and intracellular Ca^2+^ accumulation leading to endothelial swelling. During reperfusion, ROS generation and microglial activation trigger the release of pro-inflammatory cytokines such as IL-6 and TNF-α, resulting in oxidative stress, BBB disruption, neuronal injury, and subsequent cell death.

The ischemia–reperfusion injury cascade and its associated histopathological outcomes are summarized in Figure 4, which illustrates sequential cellular and vascular events including reactive oxygen species generation, glial activation, cytokine release, blood–brain barrier disruption, and neuronal injury.

Neurovascular unit-specific cellular responses to ischemia–reperfusion injury exhibit marked heterogeneity. Neurons are particularly vulnerable to oxidative stress and excitotoxic signaling, undergoing apoptotic or necrotic cell death depending on injury severity. Mitochondrial dysfunction, cytochrome c release, and caspase activation constitute key pathways mediating neuronal apoptosis. Astrocytes respond through reactive gliosis, which may confer partial neuroprotection via antioxidant mechanisms but can also exacerbate inflammatory signaling through cytokine release. Microglial activation is characterized by a shift toward a pro-inflammatory phenotype, amplifying local inflammatory responses and secondary neuronal injury. Concurrent endothelial dysfunction is associated with increased expression of adhesion molecules such as ICAM-1 and VCAM-1, facilitating leukocyte adhesion and transmigration and further compromising blood–brain barrier integrity [180,181,182].

**Figure 5:**
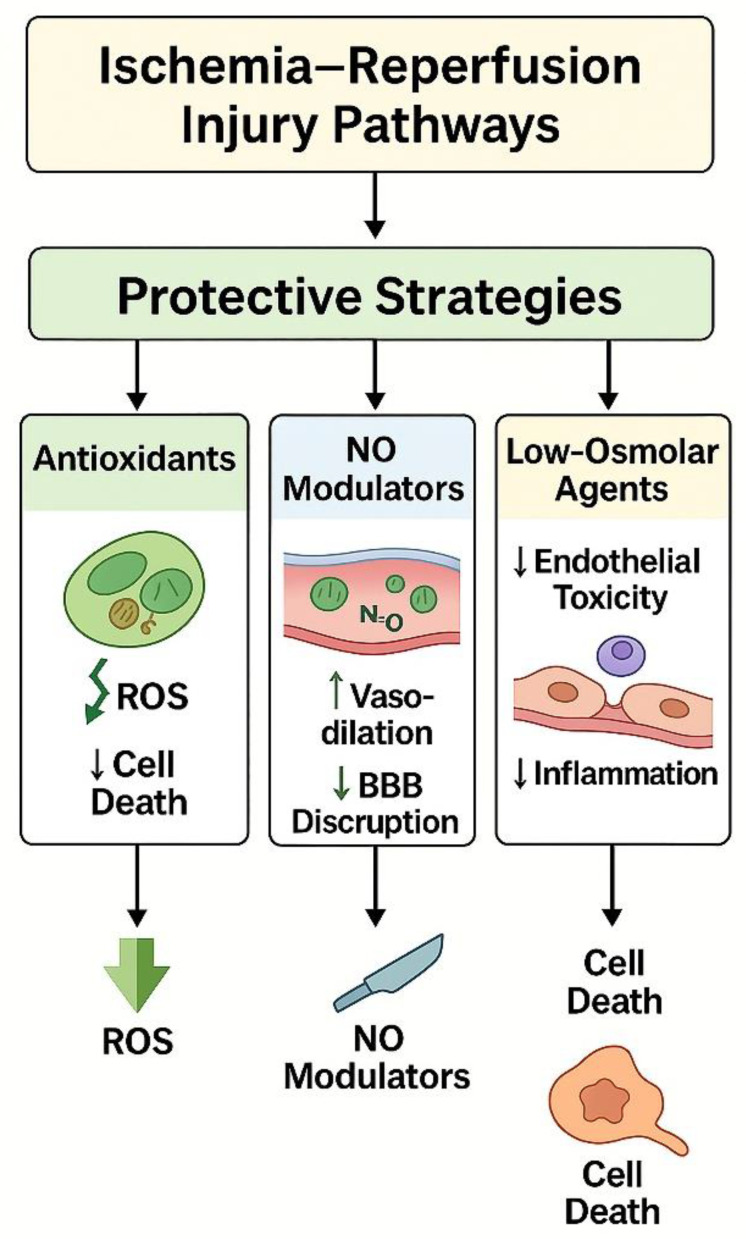
Protective Strategies and Pharmacological Interventions. Schematic illustration depicting histological and molecular mechanisms of protection against ischemia–reperfusion–related vascular injury following cerebral angiography. The figure highlights three principal therapeutic strategies: (1) Antioxidants (green)—scavenge ROS and preserve mitochondrial and endothelial integrity; (2) NO Modulators (blue)—maintain endothelial tone, promote vasodilation, and prevent BBB disruption; and (3) Low-Osmolar Contrast Agents (light gray/yellow)—reduce endothelial toxicity, inflammation, and osmotic stress. Together, these interventions attenuate oxidative stress and inflammatory responses, ultimately preserving vascular homeostasis and preventing cellular injury.

Understanding cellular responses to ischemia–reperfusion injury is essential for the development of neuroprotective strategies in the context of cerebral angiographic procedures. Experimental and clinical studies suggest that targeting oxidative stress, inflammatory signaling, excitotoxic pathways, and mitochondrial dysfunction may attenuate secondary tissue injury. Approaches aimed at modulating these processes have demonstrated potential in reducing neuronal damage and vascular dysfunction, although their clinical applicability remains under investigation. The complex pathophysiological cascade associated with ischemia–reperfusion injury and its potential therapeutic targets are summarized in Figure 5, highlighting key pathways involved in angiography-related cerebral injury.

### 7.2. Apoptosis and Necrosis Mechanisms

Cellular injury following cerebral angiography is mediated predominantly through apoptotic and necrotic pathways, resulting in distinct histopathological alterations within endothelial, neuronal, and glial cell populations. Apoptotic cell death is closely associated with oxidative stress, inflammatory signaling, and mitochondrial dysfunction, and is most frequently observed in neurons and vascular endothelial cells.

Histopathological features of apoptosis include cellular shrinkage, chromatin condensation, nuclear fragmentation, and apoptotic body formation. Activation of the intrinsic mitochondrial pathway, characterized by cytochrome c release and subsequent caspase activation, represents a central mechanism underlying angiography-associated apoptotic injury. Ultrastructural analysis demonstrates preserved cellular membranes with focal blebbing, consistent with regulated cell death processes and limited secondary inflammation [183,184,185,186].

**Figure 6:**
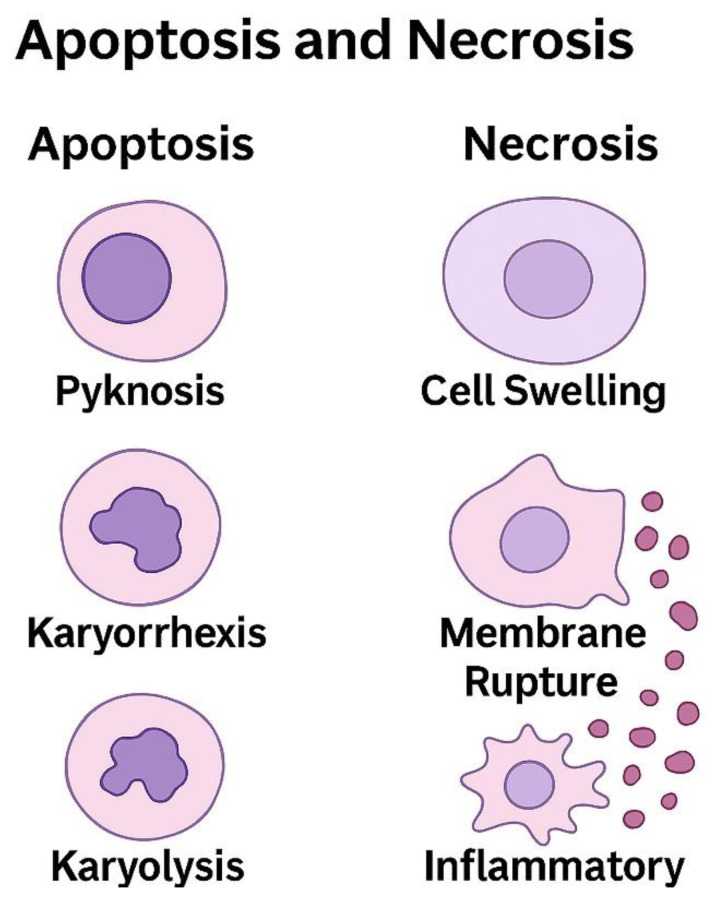
Histopathological Patterns of Apoptosis and Necrosis. Representative schematic illustration comparing the histological features of apoptotic and necrotic cell death in neural and vascular tissues following cerebral angiography. The left panel shows apoptosis, characterized by cell shrinkage, chromatin condensation (pyknosis), nuclear fragmentation (karyorrhexis), and formation of apoptotic bodies, with preserved membrane integrity and absence of inflammation. The right panel depicts necrosis, demonstrating cell swelling, membrane rupture, nuclear fading (karyolysis), cytoplasmic eosinophilia, and marked inflammatory cell infiltration. Distinct histopathological profiles highlight the controlled, non-inflammatory nature of apoptosis versus the destructive and pro-inflammatory progression of necrosis.

The principal apoptotic and necrotic pathways implicated in cerebral angiography–related cellular injury, together with their characteristic histological features, are illustrated in Figure 6.

Necrotic cell death following cerebral angiography is associated with severe energy depletion, hypoxia, and toxic cellular injury. Histopathologically, necrosis is characterized by cellular swelling, loss of membrane integrity, cytoplasmic eosinophilia, and nuclear dissolution, accompanied by release of intracellular contents into the extracellular space. This process elicits a pronounced inflammatory response, with activation of microglia and macrophages, tissue edema, and infiltration of inflammatory cells [187,188].

Inflammatory forms of necrosis, including pyroptosis, have also been implicated in angiography-related tissue injury. Pyroptosis is mediated by activation of inflammatory caspases and pore-forming pathways that result in membrane rupture and cytokine release, thereby amplifying local inflammatory responses. In addition, ferroptosis, an iron-dependent form of regulated necrosis, has been observed under conditions of excessive oxidative stress and impaired antioxidant defense. Histopathological features of ferroptosis include mitochondrial structural abnormalities and lipid peroxidation–related membrane damage, particularly in settings of blood–brain barrier disruption and contrast-induced oxidative injury [189,190].

In ischemic and hypoxic regions, apoptotic and necrotic pathways frequently coexist, resulting in mixed patterns of cellular injury. Neuronal degeneration, glial activation, and endothelial cell loss contribute to perivascular edema, inflammatory amplification, and microvascular dysfunction. Together, these regulated and unregulated cell death mechanisms underscore the complexity of angiography-associated tissue injury at the cellular level.

### 7.3. Microglial Activation and Neuroinflammation

Microglial activation represents a central component of neuroinflammatory responses associated with cerebral angiography–related injury. Depending on the nature and severity of the stimulus, activated microglia may adopt either a pro-inflammatory or a reparative phenotype. Pro-inflammatory microglial activation is characterized by increased release of cytokines such as TNF-α, IL-1β, and IL-6, accompanied by enhanced oxidative stress and nitric oxide production. This response is commonly triggered by blood–brain barrier disruption, contrast-induced toxicity, ischemia–reperfusion injury, and oxidative stress, contributing to neuronal damage and amplification of neuroinflammation [191,192,193].

**Figure 7:**
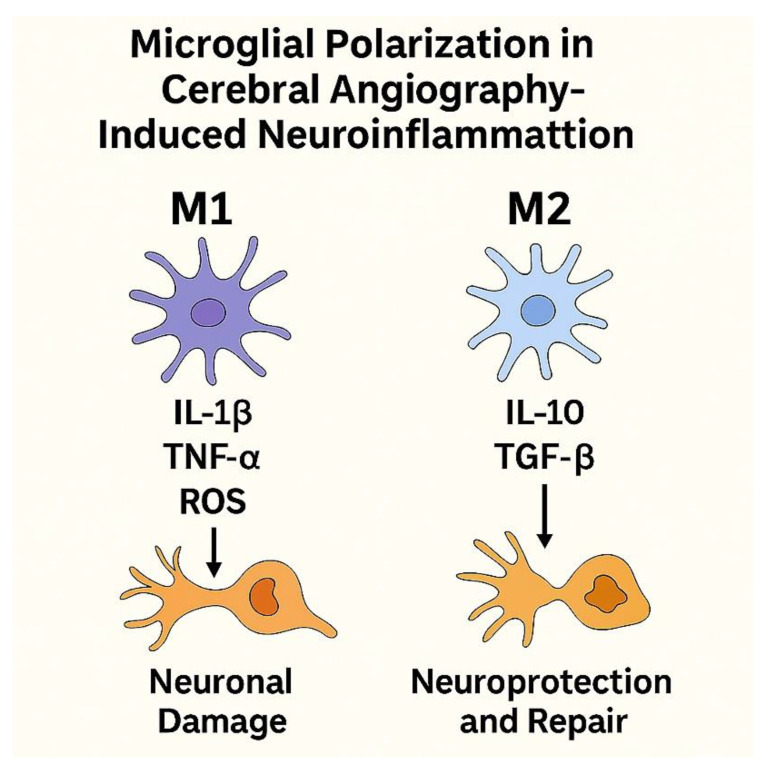
Microglial Polarization in Cerebral Angiography-Induced Neuroinflammation. Schematic representation illustrating the dual roles of microglial activation following cerebral angiography. The left panel shows the M1 phenotype (pro-inflammatory), characterized by the release of TNF-α, IL-1β, IL-6, and ROS, contributing to neuronal damage, oxidative stress, and BBB disruption. The right panel depicts the M2 phenotype (anti-inflammatory), which secretes IL-10 and TGF-β, promotes debris clearance, tissue repair, and neuroprotection. The dynamic balance between M1 and M2 polarization determines the outcome of neuroinflammation and vascular integrity in post-angiographic brain tissue.

In contrast, reparative microglial responses are associated with resolution of inflammation, clearance of cellular debris, and support of neuronal survival. The balance between these opposing activation states influences the extent and persistence of neuroinflammatory injury following cerebral angiographic procedures. The principal pathways involved in microglial activation and their contribution to angiography-associated neuroinflammation are summarized in Figure 7.

## 8. Experimental Studies: Histopathological Vascular Findings

Animal models have provided critical insights into the vascular and histopathological effects of angiographic procedures, particularly with respect to contrast-induced endothelial injury and vascular remodeling. Experimental studies consistently demonstrate endothelial dysfunction following angiography, attributed primarily to the cytotoxic and oxidative effects of contrast agents on the vascular endothelium. These alterations are associated with impaired nitric oxide bioavailability, dysregulation of vascular tone, and increased thrombogenic potential, often accompanied by inflammatory activation of the vessel wall [194,195].

Increased vascular permeability represents another prominent finding in animal models after angiography. Disruption of endothelial barrier integrity facilitates extravasation of plasma components, inflammatory cells, and cytokines, contributing to perivascular edema and tissue inflammation. Histological analyses frequently reveal structural changes within the vessel wall, including intimal and medial thickening, endothelial apoptosis, and early vascular remodeling, particularly in larger-caliber arteries exposed to higher contrast concentrations [195,196].

Vascular smooth muscle cells also exhibit adaptive and pathological responses following angiographic injury. Proliferation and migration of smooth muscle cells contribute to intimal hyperplasia and progressive vascular remodeling, which may predispose to luminal narrowing and altered blood flow. In the microvasculature, angiographic procedures have been associated with capillary rarefaction and aberrant angiogenic responses, potentially compromising tissue perfusion and microvascular integrity [197,198,199].

In addition to cerebrovascular effects, animal studies have demonstrated organ-specific vascular injury following angiography, including renal microvascular dysfunction consistent with contrast-induced nephrotoxicity. These changes are characterized by increased oxidative stress, inflammatory infiltration, and activation of cell death pathways within renal tissues, providing mechanistic support for clinically observed contrast-induced organ injury [197,198,199,200].

Collectively, findings from animal models underscore the capacity of angiographic procedures to induce endothelial dysfunction, vascular permeability alterations, inflammatory activation, and structural remodeling at both macrovascular and microvascular levels. These experimental observations provide a mechanistic framework for understanding angiography-associated vascular injury and for guiding the development of safer imaging strategies.

## 9. Conclusions and Future Directions

Cerebral angiography remains an indispensable diagnostic and interventional tool in the management of cerebrovascular disease; however, accumulating experimental and clinical evidence demonstrates that angiographic procedures can induce endothelial dysfunction and histopathological vascular injury. Endothelial damage, vascular inflammation, oxidative stress, and structural remodeling collectively contribute to angiography-associated vascular vulnerability and may predispose to both acute and long-term complications.

The findings summarized in this review underscore the central role of endothelial integrity in maintaining vascular homeostasis during and after angiographic procedures. Histopathological alterations, including endothelial cell injury, increased vascular permeability, inflammatory activation, and microvascular remodeling, highlight the importance of understanding cellular responses to contrast exposure and procedural stress. These mechanisms represent key targets for improving the safety profile of cerebral angiography.

Future directions should focus on strategies aimed at minimizing angiography-induced vascular injury while preserving diagnostic and therapeutic efficacy. Advances in contrast agent design with improved biocompatibility, optimization of procedural techniques to reduce endothelial trauma, and refinement of imaging protocols may collectively reduce vascular risk. In parallel, emerging insights into endothelial biology support the exploration of targeted protective approaches aimed at preserving endothelial function and limiting inflammatory and oxidative injury.

Technological innovations, including advanced angiographic imaging, real-time hemodynamic assessment, and integration of artificial intelligence-based image analysis, hold promise for improving procedural precision and minimizing vascular stress [55,56,65,66]. In addition, translational research exploring endothelial-protective pharmacological strategies and regenerative approaches may further enhance vascular recovery following angiographic procedures [99,100,101,124,125,171,172,173,174,175].

**Figure 8:**
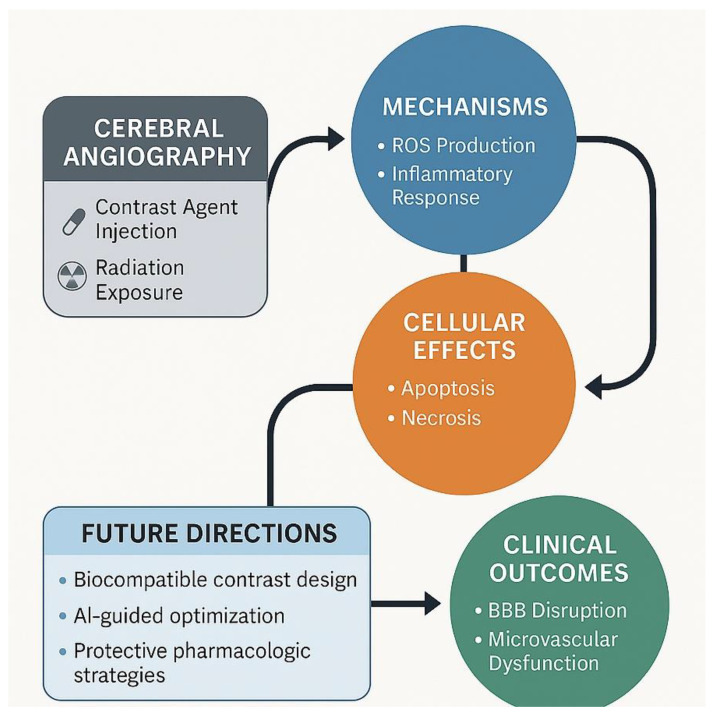
Graphical Summary of Proposed Pathophysiological Mechanisms and Future Directions. Schematic representation summarizing the proposed pathophysiological mechanisms and future strategies related to cerebral angiography–induced endothelial and histopathological changes. The diagram illustrates the sequential process beginning with cerebral angiography and contrast agent exposure, leading to endothelial activation, oxidative stress, inflammation, apoptosis, and subsequent BBB disruption and microvascular dysfunction. The figure also highlights potential future directions, including the development of biocompatible contrast agents, AI-guided optimization of angiographic procedures, and pharmacological strategies aimed at endothelial protection and vascular preservation.

The proposed mechanisms underlying angiography-induced vascular injury and potential future protective strategies are summarized in Figure 8. A multidisciplinary approach integrating imaging science, vascular biology, and bioengineering will be essential to advance safer and more effective neuroendovascular practice in the coming years [174,175,176,177].

## Figures and Tables

**Table 1:** Summary of Literature Selection Process (PRISMA 2020 Framework).

Stage	Description	Number of Records (n)	Notes/Criteria
**Identification**	Records identified through database searching (PubMed, Scopus, Web of Science)	**1142**	Initial comprehensive search using keywords related to cerebral angiography, endothelial injury, and histopathology.
**Duplicate Removal**	Duplicate studies removed	**216**	Removed identical records detected across multiple databases.
**Screening**	Titles and abstracts screened for relevance	**926**	Excluded clearly unrelated topics (non-vascular, animal-only, non-histological studies).
**Exclusion at Screening Stage**	Records excluded after initial title/abstract screening	**614**	Excluded due to irrelevance or insufficient data.
**Eligibility Assessment**	Full-text articles assessed for eligibility	**312**	Evaluated for inclusion based on relevance to cerebrovascular endothelial pathology.
**Full-Text Exclusion**	Full-text articles excluded (irrelevant, non-cerebrovascular, or no histopathological data)	**112**	Eliminated studies lacking histological or endothelial data.
**Included in Qualitative Synthesis**	Studies meeting inclusion criteria	**200**	Final set of articles included in review and discussion.

**Table 2:** Comparative Analysis: DSA vs. Other Vascular Imaging Modalities.

Modality	Invasiveness	Radiation Exposure	Contrast Requirement	Best for
**DSA**	Invasive	High	Iodinated contrast	Real-time vascular interventions, high-resolution vessel imaging
**CT Angiography (CTA)**	Non-invasive	Moderate	Iodinated contrast	Rapid vascular assessment, aortic pathologies, pulmonary embolism
**MR Angiography (MRA)**	Non-invasive	None	Gadolinium-based contrast (optional)	Soft tissue and vascular imaging without radiation
**Ultrasound Doppler**	Non-invasive	None	No contrast needed	Bedside vascular assessment, deep vein thrombosis

**Table 3:** The key differences among angiographic modalities.

Modality	Invasiveness	Radiation Exposure	Contrast Requirement	Best for
**MRA (TOF, PC, CE)**	Non-invasive	None	Optional (Gadolinium in CE-MRA)	Screening and diagnosis of cerebrovascular diseases
**DSA**	Invasive	High	Iodinated contrast	Interventional procedures, high-resolution vessel imaging
**CTA**	Non-invasive	Moderate	Iodinated contrast	Rapid assessment of aortic and extracranial vascular pathologies
**Ultrasound Doppler**	Non-invasive	None	No contrast	Hemodynamic assessment, bedside vascular evaluation

**Table 4:** Summary of Histopathological Effects and Complications.

Intervention	Histopathological Effects	Complications
**Stenting**	Endothelial damage, neointimal hyperplasia, inflammatory cell infiltration, smooth muscle cell proliferation, potential restenosis.	In-stent restenosis, late thrombosis, neoatherosclerosis, chronic inflammation.
**Balloon Angioplasty**	Endothelial disruption, medial dissection, thrombus formation, smooth muscle cell activation, extracellular matrix remodeling.	Elastic recoil, restenosis, thrombosis, vessel rupture in severe cases.
**Coil Embolization**	Thrombus formation, fibroblast infiltration, endothelialization over occlusion site, potential aneurysm recanalization.	Incomplete occlusion, recanalization, chronic inflammation, aneurysm recurrence.

**Table 5:** Cellular and Molecular Changes After Mechanical Thrombectomy.

Cellular Process	Description
**Endothelial Injury and Vascular Remodeling**	Mechanical damage to the endothelium leading to platelet aggregation, thrombin activation, local inflammation, and vascular healing via EPCs.
**Inflammatory Response and Immune Activation**	Increased IL-6, TNF-α, IL-1β levels trigger leukocyte recruitment, exacerbating BBB disruption and increasing the risk of hemorrhagic transformation.
**I/R Injury and Oxidative Stress**	Reperfusion leads to ROS overproduction, mitochondrial dysfunction, neuronal apoptosis, and increased BBB permeability, worsening neuroinflammation.
**Astrocyte and Microglial Activation**	Activated astrocytes and microglia mediate neuroprotection or neurotoxicity; excessive activation contributes to neuroinflammation and impaired plasticity.
**Neuronal and Synaptic Changes**	Prolonged ischemia results in neuronal apoptosis, excitotoxicity, and synaptic remodeling; excessive microglial pruning may hinder functional recovery.
**Blood–Brain Barrier Dysfunction**	Endothelial damage, cytokine release, and oxidative stress increase BBB permeability, leading to vasogenic edema and hemorrhagic transformation.

**Table 6:** Summary of Restenosis and Tissue Regeneration Following Mechanical Thrombectomy.

Process	Description
**Restenosis**	Re-narrowing of the vessel lumen due to endothelial dysfunction, smooth muscle cell proliferation, and extracellular matrix remodeling.
**Endothelial Injury**	Mechanical disruption exposes the subendothelial matrix, promoting platelet adhesion, activation, and an imbalance in vasoactive factors.
**Inflammatory Response**	IL-6, TNF-α, IL-1β recruit immune cells, leading to chronic inflammation, smooth muscle cell migration, and neointimal hyperplasia.
**Extracellular Matrix Remodeling**	Increased deposition of collagen and fibronectin stiffens the vascular wall, while MMPs regulate fibrosis and repair.
**Thrombogenicity**	Endothelial damage increases thrombus formation and residual platelet activation, leading to a higher risk of reocclusion.
**Endothelial Regeneration**	EPCs migrate to the injury site, and VEGF/bFGF promote neovascularization and vessel stabilization.
**Macrophage Polarization**	Shift from pro-inflammatory M1 phenotype to reparative M2 phenotype, reducing excessive inflammation and fibrosis.
**Neurovascular Remodeling**	Astrocytes and pericytes stabilize the blood–brain barrier, while synaptic plasticity and axonal sprouting improve recovery.
**Therapeutic Strategies**	Targeting inflammation, endothelial cell therapies, and bioengineered stents may reduce restenosis and enhance recovery.

## Data Availability

No new data were created.

## Supplementary Materials

The following supporting information can be downloaded at: https://www.mdpi.com/article/10.3390/jcm15030974/s1, Figure S1: jcm-15-00974-PRISMA_2020_checklist, Figure S2: PRISMA_2020_flow_diagram_new_SRs_v1.

## Abbreviations

The following abbreviations are used in this manuscript:

AVMs	Arteriovenous Malformations
DSA	Digital Subtraction Angiography
BBB	Blood–Brain Barrier
eNOS	Endothelial Nitric Oxide Synthase
NF-κB	Nuclear Factor Kappa B
ROS	Reactive Oxygen Species
TNF-α	Tumor Necrosis Factor-Alpha
IL-6	Interleukin-6
IL-1β	Interleukin-1 Beta
NO	Nitric Oxide
VE-cadherin	Vascular Endothelial Cadherin
PECAM-1	Platelet Endothelial Cell Adhesion Molecule-1
MDA	Malondialdehyde
TEM	Transmission Electron Microscopy
DNA	Deoxyribonucleic Acid
ICAM-1	Intercellular Adhesion Molecule-1
VCAM-1	Vascular Cell Adhesion Molecule-1
EPCs	Endothelial Progenitor Cells
ICG	Indocyanine Green
NIR	Near-Infrared
CE-MRA	Contrast-Enhanced Magnetic Resonance Angiography
PC	Phase-Contrast
TOF	Time-of-Flight
CE	Contrast-Enhanced
CTA	Computed Tomography Angiography
CT	Computed Tomography
MDCT	Multidetector Computed Tomography
CIN	Contrast-Induced Nephropathy
CIE	Contrast-Induced Encephalopathy
AI	Artificial Intelligence
I/R	Ischemia–Reperfusion
I/R injury	Ischemia–Reperfusion Injury
ATP	Adenosine Triphosphate
NMDA	N-Methyl-D-Aspartate
AMPA	α-Amino-3-hydroxy-5-methyl-4-isoxazolepropionic Acid
M1	Pro-inflammatory Microglial Phenotype
M2	Anti-inflammatory Microglial Phenotype
MMPs	Matrix Metalloproteinases
TGF-β	Transforming Growth Factor-Beta
VEGF	Vascular Endothelial Growth Factor
VSMCs	Vascular Smooth Muscle Cells
bFGF	Basic Fibroblast Growth Factor
TOF-MRA	Time-of-Flight Magnetic Resonance Angiography
dAVFs	Dural Arteriovenous Fistulas
DECT	Dual-Energy Computed Tomography
7T MRA	7 Tesla Magnetic Resonance Angiography
3T MRA	3 Tesla Magnetic Resonance Angiography
γ-H2AX	Phosphorylated Histone H2A Variant X (DNA damage marker)
CD34	Cluster of Differentiation 34
